# Pyroptosis-related genes *GSDMB*, *GSDMC*, and *AIM2* polymorphisms are associated with risk of non-small cell lung cancer in a Chinese Han population

**DOI:** 10.3389/fgene.2023.1212465

**Published:** 2023-06-09

**Authors:** Xia Zhang, Rongfeng Liu

**Affiliations:** ^1^Department of Respiratory Medicine, Shanxi Province Cancer Hospital/Shanxi Hospital Affiliated to Cancer Hospital, Chinese Academy of Medical Sciences/Cancer Hospital Affiliated to Shanxi Medical University, Taiyuan, Shanxi, China; ^2^Department of Medical Oncology, The Fourth Hospital of Hebei Medical University, Shijiazhuang, Hebei, China

**Keywords:** non-small cell lung cancer (NSCLC), pyroptosis, gasdermin (GSDM), absent in melanoma 2 (AIM2), polymorphisms

## Abstract

**Background:** Pyroptosis is essential for the remodeling of tumor immune microenvironment and suppression of tumor development. However, there is little information available about pyroptosis-related gene polymorphisms in non-small cell lung cancer (NSCLC).

**Methods:** Six SNPs in the *GSDMB*, *GSDMC*, and *AIM2* were genotyped in 650 NSCLC cases and 650 healthy controls using a MassARRAY platform.

**Results:** Minor alleles of rs8067378, rs2305480, and rs77681114 were associated with a lower risk of NSCLC (*p* < 0.005), whereas rs2290400 and rs1103577 were related to an increased risk (*p* < 0.00001). Moreover, rs8067378-AG/GG, rs2305480-GA/AA, and rs77681114-GA/AA genotypes were associated with a decrease in NSCLC risk (*p* < 0.005). In contrast, the TC/CC genotypes of rs2290400 and rs1103577 were associated with an elevated NSCLC risk (*p* < 0.0001). Based on the analysis of genetic models, minor alleles of rs8067378, rs2305480 and rs77681114 were related to reduced risk of NSCLC (*p* < 0.05); whereas rs2290400 and rs1103577 were related to increased risk (*p* < 0.01).

**Conclusion:** Our findings provided new insights into the roles of pyroptosis-related genes in NSCLC, as well as new factors to be considered for assessing the risk of developing this cancer.

## Introduction

The incidence and mortality of lung cancer are stubbornly high in spite of the great effort put into the related field, with approximately 1.3 million deaths worldwide each year (Siegel et al., 2021). Nearly 85% of all lung cancer patients were diagnosed with non-small cell lung cancer (NSCLC) (Jonna and Subramaniam, 2019), including the following three pathological types (Wu et al., 2021): most of lung adenocarcinoma originates from the bronchial mucosal epithelium, squamous cell carcinoma mostly originates in the larger bronchi, and large cell carcinoma often occurs in the upper lobe of the lung (Rodriguez-Canales et al., 2016). Although there are many ways to treat lung cancer, including surgery, radiotherapy, targeted drugs and chemotherapy, lung cancer is still a major challenge to human health and life around the world because of its high metastasis, high recurrence and low cure (Jones and Baldwin, 2018). According to statistics, approximately 40% and 60% of patients with stage I and II NSCLC still die from distant metastases within 5 years in patients undergoing tumor resection surgery (Torre et al., 2016). Therefore, early detection and prevention of NSCLC are crucial for improving the survival rate of the patients. Investigation of single-nucleotide polymorphisms (SNPs) in driver genes has proven to be a potential strategy to elaborate the hereditary susceptibility to NSCLC (Feng et al., 2020; Luo et al., 2021). Combination of the SNPs strategy and nowadays tumor related research hotspot might generate novel significative genotyping data and provide theoretical basis for early prevention of the disease.

Pyroptosis was first proposed to describe the process of programmed cell death caused by *Salmonella* infection of macrophages leading to their inflammatory death (D’Souza and Heitman, 2001). Pyroptosis can protect cells from infection by eliminating pathogen host cells and triggering an inflammatory response, with the symptoms of cell swelling, nuclear clotting, membranolysis, and the secretion of inflammatory cytokines and damage-related molecular patterns (Man and Kanneganti, 2016). Pyroptosis has been considered as a Caspase-1/11-induced programmed cell death, but the specific mechanism of Caspase-induced pyroptosis has been studied for a long time until the role of gasdermin (GSDM) family was revealed (Case et al., 2013; Li et al., 2020). It has been found several members in GSDM family, including GSDMA/B/C/D/E and DFNB59 (Broz et al., 2020). The GSDMs could be cleaved and activated by protease and then mediating pyroptosis, the GSDMB and GSDMC were processed by Caspase-3/6/7/granzyme A and Caspase-8 into their active form, respectively (Wang et al., 2017). GSDMB is high expressed in several types of cancers, and its expression level is related with poor prognosis of patients (Li et al., 2020). Overexpression of GSDMC also has relativity with a bad outcomes of lung adenocarcinoma patients (Wei et al., 2020). Moreover, inflammasome absent in melanoma 2 (AIM2) can recruit and activate Caspase-1, subsequently enhance the release of interleukin (IL)-1β and IL-18, and finally induce the pyroptosis (Sharma et al., 2019). The high expression of AIM2 has been found in NSCLC tissues, and functioned as an oncogene by influencing the formation of inflammasome (Zhang et al., 2019). However, little study focused on the SNPs in *GSDMB*, *GSDMC*, and *AIM2* among patients with NSCLC.

Considering the above research background, we finally focused on six SNPs in *GSDMB*, *GSDMC*, and *AIM2* based on previous studies. The rs8067378 (Li et al., 2022) and rs2305480 (Karunas et al., 2021) in *GSDMB* were found to be protective SNPs for cervical squamous intraepithelial lesion and asthma, respectively; while *GSDMB*-rs2290400 was correlated with asthma combined with allergic rhinitis (Karunas et al., 2021). Moreover, *GSDMC*-rs77681114 was related to reduced risk of lumbar disc herniation (Wu et al., 2020). In addition, *AIM2*-rs1103577 has been found protective role on risk of tuberculosis (Figueira et al., 2021), and rs2298803 in *AIM2* was investigated in patients with rectal cancer and have no correlation with adverse events of postoperative chemoradiotherapy (Chen et al., 2023). We distinguished the alleles and genotypes of these SNPs in our study cohort, and made a disease risk prediction using genetic model analysis.

## Materials and methods

### Participants

We enrolled 650 patients with histopathologically diagnosed NSCLC and 650 healthy controls for this case-control study. Each of the participants was recruited from Shanxi Province Cancer Hospital. There were no previous treatments for any of the cases, and all were newly diagnosed. Blood donors without a history of cancer, immune disorders, or serious diseases were used as controls. We obtained written informed consent from each subject, and the study was approved by the Ethics Department of Shanxi Province Cancer Hospital and was carried out in accordance with the World Medical Association Declaration of Helsinki: Ethical Principles for Medical Research Involving Human Subjects.

### Genotyping

Five milliliters of whole blood was collected from each subject in tubes containing ethylenediaminetetraacetic acid. DNA was extracted using a QIAamp DNA Blood Midi Kit (QIAGEN, Germany). Spectrometry (DeNovix DS-11FX Ultramicro spectrophotometer, United States) was used to measure the DNA concentration. Primers were designed using Sequenom MassARRAY Assay Design 3.0 software. The primers used for this study is listed in Table 1. SNP genotyping was performed on a Mass ARRAY iPLEX platform (Sequenom, San Diego, CA, United States) according to the manufacturer’s instructions. Assay design and mass spectrometric genotyping were performed as previously described (Gabriel et al., 2009).

**TABLE 1:** PCR primers used for this study.

SNP	1st-PCR primer sequences	2nd-PCR primer sequences	UEP sequences
rs8067378	ACG​TTG​GAT​GCT​GTG​AGT​GGA​AAG​CTT​GAC	ACG​TTG​GAT​GAC​CTG​GCA​GTG​ATA​TAA​ACG	GATATAAACGTTTTTCCC
rs2305480	ACG​TTG​GAT​GCT​AGG​TAT​CTG​AGG​TCC​TGA	ACG​TTG​GAT​GAA​AAG​GCT​GCT​TAG​GAG​AGG	AGGAGAGGCTTGTCTG
rs2290400	ACG​TTG​GAT​GGT​TTT​CCA​GTC​TCA​GAA​GCG	ACG​TTG​GAT​GTA​AGG​ATC​TCA​GGG​CCT​TAC	CTCCCACTGACTCTT
rs77681114	ACG​TTG​GAT​GCC​CAT​TAT​GGC​TTC​AAG​GAG	ACG​TTG​GAT​GCC​TAA​AGA​AAC​TTC​AAC​AGG	TTCAACAGGATTCAAA
rs1103577	ACG​TTG​GAT​GTA​CTT​CCA​CTA​CCT​ATC​CCC	ACG​TTG​GAT​GGA​TGA​TTC​CCG​GCT​TTC​TG	CTTTCTGGCTTGAGC
rs2298803	ACG​TTG​GAT​GGT​CCT​CTG​CTA​GTT​AAG​CTC	ACG​TTG​GAT​GAG​CTC​CTC​TAT​GGT​GCT​TAC	TGGTGCTTACCTCCTGA

### Statistical analyses

The statistical analyses were carried out using SPSS package version 20.0 (SPSS, Chicago, IL, United States). The chi-square test was used to compare the gender and smoking status, and the Student t-test was used to compare the age between cancer patients and healthy subjects, respectively. Controls were checked for deviations from Hardy-Weinberg equilibrium (HWE) by measuring minor allele frequencies (MAFs). SNPstats (https://www.snpstats.net/start.htm) was used to evaluate the associations between SNPs and NSCLC risk, and the results are presented in odds ratios (ORs) and 95% confidence intervals (CIs) with adjustments for sex, age and smoking status. Statistical significance was established when *p* < 0.05.

## Results

Table 2 shows the sex, age, and smoking status of the participants. Sex, age, and smoking status did not differ significantly between case and control groups (*p* > 0.05). Adenocarcinoma, squamous cell carcinoma, adenosquamous carcinoma, and large cell lung cancer account for 50.5%, 41.1%, 4.4%, and 4.0% of NSCLC cases, respectively.

**TABLE 2:** The demographic characteristics of the participants.

Characteristics	Case (*n* = 650)	Control (n = 650)	χ^2^/t	*p*
Sex (%)				
Male	426 (65.5)	403 (62.0)	1.611	0.204
Female	224 (34.5)	247 (38.0)	0.934	0.350
Age				
mean ± SD	56.98 ± 10.17	56.45 ± 10.25	1.603	0.205
Smoking (%)				
Yes	423 (65.1)	400 (61.5)		
No	227 (34.9)	250 (38.5)		
Pathological types				
Adenocarcinoma	328 (50.5)			
Squamous cell carcinoma	267 (41.1)			
Adenosquamous carcinoma	29 (4.4)			
Large cell lung cancer	26 (4.0)			
Tumor staging				
I or II	236 (36.3)			
III or IV	414 (63.7)			

The gene location information of candidate SNPs and their MAFs in cases and controls are listed in Table 3. Rs2305480 was a missense variant and led to a changed amino acid Pro > Ser, rs77681114 was a synonymous variant (Asn > Asn), other SNPs were intron, downstream or non-coding transcript variant. All SNPs matched HWE (*p* > 0.05). Three beneficial SNPs, rs8067378, rs2305480 and rs7768114, were significantly associated with a significantly lower risk of NSCLC after comparing the MAFs between cases and controls (rs8067378: OR = 0.666, 95% CI: 0.548–0.810, *p* = 0.00004; rs2305480: OR = 0.663, 95% CI: 0.549–0.802, *p* = 0.00002; rs77681114: OR = 0.751, 95% CI: 0.617–0.913, *p* = 0.00401). As well, two additional SNPs, rs2290400 and rs1103577, were linked to increased NSCLC risk (rs2290400: OR = 1.540, 95% CI: 1.296–1.830, *p* < 0.00001; rs1103577: OR = 1.497, 95% CI: 1.263–1.774, *p* < 0.00001).

**TABLE 3:** The MAF and HWE of candidate SNPs between NSCLC cases and healthy controls.

SNP	Gene	Position	Allele	Region	MAF-case	MAF-control	HWE *p*	OR (95% CI)	*p*
rs8067378	GSDMB	chr17:39895095	A>G	Downstream Variant	0.17	0.23	0.91	0.666 (0.548–0.810)	0.00004*
rs2305480	GSDMB	chr17:39905943	G>A	Missense Variant	0.18	0.25	0.25	0.663 (0.549–0.802)	0.00002*
rs2290400	GSDMB	chr17:39909987	T>C	Intron Variant	0.33	0.24	0.67	1.540 (1.296–1.830)	<0.00001*
rs77681114	GSDMC	chr8:129750045	G>A	Synonymous Variant	0.17	0.22	0.1	0.751 (0.617–0.913)	0.00401*
rs1103577	AIM2	chr1:159130525	T>C	Intron Variant	0.34	0.26	0.081	1.497 (1.263–1.774)	<0.00001*
rs2298803	AIM2	chr1:159076640	T>C	Non Coding Transcript Variant	0.33	0.31	0.2	1.080 (0.917–1.274)	0.35622

SNP, single nucleotide polymorphism; MAF, minor allele frequency; HWE, Hardy–Weinberg equilibrium.

*p* < 0.05 indicates statistical significance.

Table 4 shows the genotype frequencies of candidate SNPs. It was considered that the wild type genotype was the reference genotype. On the basis of the genotype frequencies of SNPs in cases and controls, the OR and 95% confidence interval were calculated for homozygous mutation genotypes and heterozygous mutation genotypes. There was a significant decrease in risk of NSCLC for the AG and GG genotypes of rs8067378 compared to the wild type AA (*p* = 0.0001). Similarly, the GA and AA genotypes of rs2305480 (*p* < 0.0001) and rs77681114 (*p* = 0.0025) were also determined to be protective genotypes for NSCLC. In contrast, the TC/CC genotypes of rs2290400 and rs1103577 were associated with different levels of elevated NSCLC risk (*p* < 0.0001).

**TABLE 4:** Genotype frequency distributions between NSCLC cases and healthy controls.

SNP	Genotype	Control	Case	OR (95% CI)	*p*
rs8067378	AA	385 (59.2%)	454 (69.8%)	1	0.0001*
	AG	232 (35.7%)	177 (27.2%)	0.63 (0.50–0.80)	
	GG	33 (5.1%)	19 (2.9%)	0.47 (0.26–0.85)	
rs2305480	GG	362 (55.7%)	433 (66.6%)	1	<0.0001*
	GA	254 (39.1%)	201 (30.9%)	0.64 (0.50–0.82)	
	AA	34 (5.2%)	16 (2.5%)	0.38 (0.21–0.70)	
rs2290400	TT	379 (58.3%)	292 (44.9%)	1	<0.0001*
	TC	232 (35.7%)	293 (45.1%)	1.64 (1.30–2.07)	
	CC	39 (6%)	65 (10%)	2.21 (1.44–3.39)	
rs77681114	GG	392 (60.3%)	435 (66.9%)	1	0.0025*
	GA	235 (36.1%)	207 (31.9%)	0.79 (0.62–1.00)	
	AA	23 (3.5%)	8 (1.2%)	0.30 (0.13–0.68)	
rs1103577	TT	351 (54%)	279 (42.9%)	1	<0.0001*
	TC	265 (40.8%)	306 (47.1%)	1.51 (1.20–1.90)	
	CC	34 (5.2%)	65 (10%)	2.49 (1.59–3.89)	
rs2298803	TT	298 (45.9%)	294 (45.2%)	1	0.220
	TC	295 (45.4%)	281 (43.2%)	0.95 (0.76–1.20)	
	CC	57 (8.8%)	75 (11.5%)	1.34 (0.91–1.96)	

SNP, single nucleotide polymorphism; OR, odds ratio; CI, confidence interval; *p* < 0.05 indicates statistical significance.

Furthermore, we introduced three classical genetic models—dominant, recessive, and log-additive—so we could better evaluate SNPs’ impact on NSCLC risk. Table 5 shows five SNPs that increase or decrease the risk of the disease. All three genetic models showed reduced risk of NSCLC for minor alleles of rs8067378, rs2305480, and rs77681114; while rs2290400 and rs1103577 showed increased risk of the disease (*p* < 0.01).

**TABLE 5:** Association between SNPs and NSCLC risk in genetic models.

SNP	Model	Genotype	Control	Case	OR (95% CI)	*p*
rs8067378	Dominant	AA	385 (59.2%)	454 (69.8%)	1	<0.0001*
		AG-GG	265 (40.8%)	196 (30.1%)	0.61 (0.49–0.77)	
	Recessive	AA-AG	617 (94.9%)	631 (97.1%)	1	0.041*
		GG	33 (5.1%)	19 (2.9%)	0.55 (0.31–0.99)	
	Log-additive	—	—	—	0.65 (0.54–0.80)	<0.0001*
rs2305480	Dominant	GG	362 (55.7%)	433 (66.6%)	1	<0.0001*
		GA-AA	288 (44.3%)	217 (33.4%)	0.61 (0.48–0.77)	
	Recessive	GG-GA	616 (94.8%)	634 (97.5%)	1	0.0076*
		AA	34 (5.2%)	16 (2.5%)	0.45 (0.25–0.83)	
	Log-additive	—	—	—	0.63 (0.52–0.78)	<0.0001*
rs2290400	Dominant	TT	379 (58.3%)	292 (44.9%)	1	<0.0001*
		TC-CC	271 (41.7%)	358 (55.1%)	1.72 (1.38–2.15)	
	Recessive	TT-TC	611 (94%)	585 (90%)	1	0.0056*
		CC	39 (6%)	65 (10%)	1.78 (1.18–2.70)	
	Log-additive	—	—	—	1.56 (1.30–1.85)	<0.0001*
rs77681114	Dominant	GG	392 (60.3%)	435 (66.9%)	1	0.013*
		GA-AA	258 (39.7%)	215 (33.1%)	0.75 (0.59–0.94)	
	Recessive	GG-GA	627 (96.5%)	642 (98.8%)	1	0.0047*
		AA	23 (3.5%)	8 (1.2%)	0.33 (0.15–0.75)	
	Log-additive	—	—	—	0.72 (0.59–0.89)	0.0023*
rs1103577	Dominant	TT	351 (54%)	279 (42.9%)	1	<0.0001*
		TC-CC	299 (46%)	371 (57.1%)	1.62 (1.30–2.03)	
	Recessive	TT-TC	616 (94.8%)	585 (90%)	1	0.0009*
		CC	34 (5.2%)	65 (10%)	2.04 (1.32–3.14)	
	Log-additive	—	—	—	1.54 (1.29–1.85)	<0.0001*
rs2298803	Dominant	TT	298 (45.9%)	294 (45.2%)	1	0.88
		TC-CC	352 (54.1%)	356 (54.8%)	1.02 (0.82–1.27)	
	Recessive	TT-TC	593 (91.2%)	575 (88.5%)	1	0.088
		CC	57 (8.8%)	75 (11.5%)	1.37 (0.95–1.97)	
	Log-additive	—	—	—	1.08 (0.91–1.28)	0.37

SNP, single nucleotide polymorphism; OR, odds ratio; CI, confidence interval; *p* < 0.05 indicates statistical significance.

Finally, the participants were divided into four subgroups according to the age and smoking status (Table 6). The rs8067378, rs2305480, and rs2290400 remained significant in each subgroup (*p* < 0.05). However, rs77681114 had no protective influence on the NSCLC in smokers (*p* > 0.05), and rs1103577 was not linked to NSCLC risk in nonsmokers (*p* > 0.05). Finally, we also looked at the connection between SNPs and risk of disease in patients with adenocarcinoma and squamous cell carcinoma, respectively (Table 7). All of the SNPs remained significant except rs2305480 (*p* < 0.05). The rs2305480 had no protective role for risk of squamous cell carcinoma (*p* > 0.05).

**TABLE 6:** Associations of candidate SNPs with NSCLC risk in four subgroups.

SNP	Model	Genotype	≥50		<50		Smokers		Nonsmokers	
			OR (95% CI)	*p*	OR (95% CI)	*p*	OR (95% CI)	*p*	OR (95% CI)	*p*
rs8067378	Dominant	AA	1		1	0.051	1	0.001*	1	0.012*
		AG-GG	0.60 (0.46–0.79)	0.0002*	0.62 (0.38–1.00)		0.62 (0.47–0.83)		0.60 (0.40–0.90)	
	Recessive	AA-AG	1		/	/	1	0.8	/	/
		GG	0.95 (0.50–1.80)	0.87	/		0.92 (0.48–1.76)		/	
	Log-additive	/	0.69 (0.55–0.86)	0.0011*	0.54 (0.35–0.82)	0.0032*	0.71 (0.56–0.90)	0.0043*	0.55 (0.38–0.79)	0.001*
rs2305480	Dominant	GG	1	0.0005*	1	<0.0001*	1	0.0013*	1	0.0001*
		GA-AA	0.62 (0.47–0.81)		0.22 (0.11–0.46)		0.57 (0.40–0.80)		0.46 (0.31–0.68)	
	Recessive	GG-GA	1	0.013*	1	0.42	1	0.039*	1	0.083
		AA	0.45 (0.24–0.86)		0.50 (0.09–2.81)		0.47 (0.22–0.98)		0.41 (0.14–1.18)	
	Log-additive	/	0.64 (0.51–0.81)	0.0001*	0.28 (0.15–0.55)	<0.0001*	0.61 (0.46–0.81)	0.0006*	0.50 (0.35–0.70)	<0.0001*
rs2290400	Dominant	TT	1	0.0019*	1	<0.0001*	1	<0.0001*	1	0.007*
		TC-CC	1.49 (1.16–1.91)		4.01 (2.34–6.88)		1.89 (1.42–2.51)		1.67 (1.15–2.42)	
	Recessive	TT-TC	1	0.64	1	<0.0001*	1	0.14	1	0.0097*
		CC	1.12 (0.69–1.83)		5.76 (2.30–14.42)		1.49 (0.88–2.53)		2.36 (1.21–4.61)	
	Log-additive	/	1.31 (1.07–1.61)	0.0075*	3.20 (2.10–4.87)	<0.0001*	1.61 (1.28–2.03)	<0.0001*	1.58 (1.19–2.10)	0.0015*
rs77681114	Dominant	GG	1	0.29	1	0.0002*	1	0.27	1	0.0031*
		GA-AA	0.86 (0.66–1.13)		0.41 (0.25–0.66)		0.84 (0.62–1.14)		0.57 (0.40–0.83)	
	Recessive	GG-GA	1	0.0066*	/	/	1	0.11	1	0.017*
		AA	0.34 (0.15–0.78)		/	/	0.43 (0.14–1.27)		0.25 (0.07–0.89)	
	Log-additive	/	0.80 (0.63–1.02)	0.072	/	/	0.82 (0.62–1.08)	0.15	0.57 (0.41–0.80)	0.0008*
rs1103577	Dominant	TT	1	0.0007*	1	0.016*	1	<0.0001*	1	0.22
		TC-CC	1.55 (1.20–1.99)		1.81 (1.11–2.93)		2.40 (1.81–3.19)		0.79 (0.55–1.15)	
	Recessive	TT-TC	1	0.0011*	1	0.4	1	0.008*	1	0.054
		CC	2.22 (1.35–3.65)		1.46 (0.59–3.61)		2.13 (1.20–3.79)		1.88 (0.98–3.61)	
	Log-additive	/	1.52 (1.24–1.86)	0.0001*	1.58 (1.07–2.35)	0.021*	2.01 (1.59–2.54)	<0.0001*	0.99 (0.74–1.32)	0.94

SNP, single nucleotide polymorphism; OR, odds ratio; CI, confidence interval; *p < 0.05 indicates statistical significance.

**TABLE 7:** Association between Candidate SNPs and risk of Adenocarcinoma and Squamous cell carcinoma.

SNP	Model	Genotype	Adenocarcinoma		Squamous cell carcinoma	
			OR (95% CI)	*p*	OR (95% CI)	*p*
rs8067378	Dominant	AA	1	0.0007*	1	0.0001*
		AG-GG	0.61 (0.46–0.82)		0.53 (0.39–0.72)	
	Recessive	AA-AG	1	0.03*	1	0.57
		GG	0.43 (0.19–0.98)		0.82 (0.40–1.66)	
	Log-additive	—	0.63 (0.49–0.82)	0.0003*	0.62 (0.47–0.81)	0.0003*
rs2305480	Dominant	GG	1	<0.0001*	1	0.67
		GA-AA	0.54 (0.40–0.72)		0.93 (0.68–1.29)	
	Recessive	GG-GA	1	0.0079*	1	0.18
		AA	0.34 (0.14–0.83)		0.61 (0.28–1.30)	
	Log-additive	—	0.56 (0.43–0.72)	<0.0001*	0.89 (0.68–1.16)	0.4
rs2290400	Dominant	TT	1	0.0002*	1	0.024*
		TC-CC	1.67 (1.27–2.19)		1.40 (1.05–1.88)	
	Recessive	TT-TC	1	0.073	1	0.0023*
		CC	1.59 (0.96–2.62)		2.24 (1.34–3.73)	
	Log-additive	—	1.49 (1.21–1.84)	0.0002*	1.43 (1.14–1.79)	0.0021*
rs77681114	Dominant	GG	1	0.0003*	1	0.14
		GA-AA	0.59 (0.44–0.79)		1.25 (0.93–1.69)	
	Recessive	GG-GA	1	0.041	1	0.012*
		AA	0.39 (0.15–1.04)		0.21 (0.05–0.92)	
	Log-additive	—	0.61 (0.47–0.79)	0.0001*	1.09 (0.83–1.44)	0.52
rs1103577	Dominant	TT	1	0.0047*	1	<0.0001*
		TC-CC	1.49 (1.13–1.96)		1.91 (1.41–2.59)	
	Recessive	TT-TC	1	0.026*	1	0.012*
		CC	1.80 (1.08–3.00)		2.03 (1.18–3.50)	
	Log-additive	—	1.43 (1.15–1.79)	0.0013*	1.70 (1.34–2.16)	<0.0001*

SNP, single nucleotide polymorphism; OR, odds ratio; CI, confidence interval; *p < 0.05 indicates statistical significance.

## Discussion

Pyroptosis is a new type of programmed cell death, which has been widely studied in various diseases in recent years, and the importance of this pathway to regulate tissue development and homeostasis has also received attention (Jia et al., 2023). Pyroptosis -related factors have a dual mechanism of promoting or inhibiting tumorigenesis, and can affect tumor progression by modulating malignant phenotypes such as cell morphology, proliferation, invasion, migration, and chemotherapy tolerance through multiple molecular signaling pathways, and may affect a patient’s prognosis (Frank and Vince, 2019). In our study, we identified five SNPs associated with increased or reduced risk of NSCLC associated with the pyroptosis-related genes *GSDMB*, *GSDMC*, and *AIM2*, which may shed light on the relationship between pyroptosis and NSCLC pathogenesis, as well as provide theoretical foundations for detecting and preventing the disease early.

*GSDMB*, located at 17q21, encodes the GSDMB that participate in pyroptosis as a key molecule. Ding’s group reported that GSDMB could be cut by Caspase-1 and released the N-terminus domain that induce cell pytoptosis (Ding et al., 2016), while Chen’s team demonstrated that GSDMB could not form pores on cytomembrane, but promoted non-classical pyroptosis through an enhancement of caspase-4 activity (Chen et al., 2019). With the research development, Chao’ lab argued that GSDMB could not be the substrate for human Caspase-1/4/5/11 due to lacking of the specific interdomain, but it could be cleaved by Caspase-3/6/7, which indicating that an apoptosis-pyroptosis cross-talk may be occurring (Chao et al., 2017). Also, lots of studies have shown that the genetic polymorphisms were linked to risk of autoimmune disease. Imraish et al. (2022) reported that *GSDMB*-rs7216389 has potential influence on IgE levels of patients with asthma in Jordanian population. Shamsi et al. (2023) reported that *GSDMB*-rs4795400, rs2305479, and rs12450091 were associated with risk of allergic rhinitis. As for cancer studies about *GSDMB* polymorphisms, Lutkowska et al. (2017) found that rs8067378 A>G variant may elevate the expression of GSDMB and increased the risk of the cervical squamous cell carcinomas in a Polish population, while Li et al. (2022) further reported that rs8067378 was a risk-reducing variant for cervical squamous intraepithelial lesion. We for the first time identified that a declined NSCLC risk was correlated with rs8067378 and rs2305480 in GSDMB, whereas an increased risk was associated with rs2290400. These results provided new evidence for the involvement of *GSDMB* in development and progression of NSCLC, while the molecular mechanism needed to be further explored. It is worth noting that rs2305480 is a missense variant and leads to Pro > Ser. Pro is a non-polar and hydrophobic amino acid, while Ser is a polar uncharged amino acid. We supposed that rs2305480 may has an effect on the progression of the disease through changing the conformation of GSDMB and its function in cell pyroptosis.

It has recently been revealed that GSDMC plays a role in cell pyroptosis as a member of the GSDM family. Hou’s group found that Caspase-8 can cut GSDMC in hypoxic breast cancer cells, and its expression level was mediated by the PD-L1, following by TNF-α induced pyroptosis (Hou et al., 2020). Moreover, Miguchi et al. established that an increase in GSDMC expression was linked to mutations in TGF-β receptor type II, and leading to a promotion of cell growth in colorectal cancer and xenograft tumor volum *in vivo* (Miguchi et al., 2016). A similar tumor-promoting role in lung adenocarcinoma was demonstrated by Wei’s Lab, upregulation of GSDMC was linked to poor outcomes, making it be a promising target for the disease (Wei et al., 2020). Furthermore, Yan’s group pointed that GSDMC functioned as an oncogene that promoting the cell proliferation and migration in pancreatic adenocarcinoma (Yan et al., 2022). In these studies, it was demonstrated that GSDMC played a crucial role in cancer development, especially in pyroptosis. However, little study focused on the genetic polymorphisms in *GSDMC*. Among Chinese Han, Wu et al. found that GSDMC-rs77681114 significantly decreased risk of lumbar disc herniation (Wu et al., 2020). We demonstrated that the variant rs77681114G>A is protective against NSCLC. However, smokers were not significantly affected by rs77681114 in a stratification analysis. We supposed that the protective role of this variant might be neutralized with cigarette smoking in NSCLC patients, while the hypothesis and detailed mechanisms should be verified and investigated in further studies.

AIM2 belongs to a family of inflammasomes and function as an intracellular DNA receptor that recognize double-stranded DNA released into the cytoplasm, activate downstream related effector proteins and induce cell pyroptosis (Wang et al., 2020). In addition to activating inflammasomes for immune function, studies have found that AIM2 also has the dual effects of promoting or inhibiting cancer development. Choubey et al. (2000) firstly reported the tumor suppressor role of AIM2, upregulation of AIM2 inhibited the cell proliferation and enhanced cell death in melanoma. Moreover, AIM2 was low expressed and played a tumor-inhibiting role in colon, liver, renal, breast and prostate cancers (Qin et al., 2022). In contrast, Farshchian et al. (2017) revealed a pro-tumorigenic role of AIM2, downregulation of AIM2 reduced the viability and invasion of cutaneous squamous cell carcinoma. Zhang et al. (2019) and Qi et al. (2020) also reported the oncogenic role of AIM2 in NSCLC through the inflammasome and modulation of mitochondrial dynamics, respectively. According to our understanding, few study focused on the AIM2 polymorphisms and cancer risk. We genotyped two SNPs, rs1103577, and rs2298803 in *AIM2*, and observed that rs1103577T>C was a risky variant for NSCLC. A subgroup analysis revealed that rs1103577T>C increased NSCLC risk in smokers but not in non-smokers, suggesting that rs1103577 may have interaction with smoking in the onset or development of NSCLC. The results provided an important detection site for early prevention of NSCLC.

In an association study, population stratification may lead to false positive or negative results. Thus, we stratified our analysis according to age and smoking status. Smokers and nonsmokers exhibited different results for GSMDC-rs77681114 and AIM2-rs1103577, suggesting that these two variants might interact with smoking in NSCLC progression. In addition, we also evaluated the association of candidate SNPs and different pathological type of NSCLC. The *GSDMB*-rs2305480 was a protective variant for adenocarcinoma, but not for squamous cell carcinoma, which could be explained by the different pathogenesis between the two pathological types of NSCLC.

Although the present study revealed the association between pyroptosis-related genes and NSCLC, there are also some potential limitations. Firstly, history of other lung diseases and family history of cancer might have associations with risk of the NSCLC; however, we have no related information to analyze, because the participants were collected in a very long time period, we did not design the factors from the very beginning. Secondly, the SNPs identified here could only represent the Chinese Han population, further validation study need to be done in other populations. Thirdly, our results needed to be further validated in functional studies.

In conclusion, we demonstrated that *GSDMB*-rs8067378, rs2305480, and *GSMDC*-rs77681114 were linked to a reduced NSCLC risk, while *GSDMB*-rs2290400 and *AIM2*-rs1103577 were related to an increased risk of the disease. Our findings provided new insights into the roles of pyroptosis-related genes in NSCLC, as well as new factors to be considered for assessing the risk of developing this cancer.

## Data Availability

The original contributions presented in the study are included in the article/supplementary material, further inquiries can be directed to the corresponding author.
